# Lymphoma and Lymphomagenesis in Primary Sjögren’s Syndrome

**DOI:** 10.3389/fmed.2018.00102

**Published:** 2018-04-13

**Authors:** Alessia Alunno, Maria Comasia Leone, Roberto Giacomelli, Roberto Gerli, Francesco Carubbi

**Affiliations:** ^1^Rheumatology Unit, Department of Medicine, University of Perugia, Perugia, Italy; ^2^Rheumatology Unit, Department of Biotechnological and Applied Clinical Sciences, University of L’Aquila, L’Aquila, Italy; ^3^ASL1 Avezzano-Sulmona-L’Aquila, Department of Medicine, L’Aquila, Italy

**Keywords:** Sjögren’s syndrome, lymphoma, salivary glands, cryoglobulins, germinal centers

## Abstract

Primary Sjögren’s syndrome (pSS) is a systemic autoimmune disease mainly affecting exocrine glands and leading to impaired secretory function. The clinical picture is dominated by signs and symptoms of mucosal dryness and the course of the disease is mild and indolent in the majority of cases. However, a subgroup of patients can also experience extraglandular manifestations that worsen the disease prognosis. pSS patients are consistently found to have a higher risk of developing non-Hodgkin lymphoma (NHL) compared with patients with other autimmune disorders and to the general population. NHL is the most severe comorbidity that can occur in pSS, therefore recent research has aimed to identify reliable clinical, serological, and histological biomarkers able to predict NHL development in these subjects. This review article encompasses the body of evidence published so far in this field highlighting the challenges and pitfalls of different biomarkers within clinical practice. We also provide an overview of epidemiological data, diagnostic procedures, and evidence-based treatment strategies for NHL in pSS.

## Introduction

Primary Sjögren’s syndrome (pSS) is an autoimmune disorder primarily characterized by chronic inflammation of exocrine glands, leading to reduced secretory capacity (1, 2). A consistent subgroup of patients also experiences extraglandular manifestations that can involve virtually any organ and system (3). The evolution into B-cell lymphoma represents one of the main causes of decreased survival in pSS and occurs in about 5% of patients (4). Lymphoid proliferation of the salivary glands is a peculiar feature of pSS and can be either reactive, including in lymphoepithelial sialadenitis (LESA), also known as myoepithelial sialadenitis (MESA), or neoplastic. LESA/MESA display a peculiar spectrum of histopathologic lesions ranging from, on one hand, a fully benign lymphoid infiltrate that is sometimes associated with lymphoid follicular structures, however, does not display immunoglobulin (Ig) light chain restriction in B cells, to lymphoproliferative lesions containing centrocyte-like cells that may present Ig light chain restriction in B cells. Therefore, lymphoepithelial lesions display a variable aggressiveness even without malignant features. About 50% of LESA/MESA include B-cell clones, but this does not correlate with morphological or clinical evidence of overt lymphoma (5, 6). Lymphomas of the salivary glands are predominantly B-cell type, including extranodal marginal zone B-cell lymphoma of the mucosa-associated lymphoid tissue (MALT). Parotid and submandibular salivary glands are the most frequent localization of MALT lymphomas in pSS, but other mucosal sites could be affected, including orbits, naso-pharynx, stomach, thyroid, and lung. During the last 40 years, a consistent number of studies have attempted to identify clinical, serological, and histological predictors of lymphoma in pSS patients, but although progress has been achieved in this field, many aspects remain to be fully elucidated.

The purpose of this review article is to provide an overview of the current knowledge of lymphoma biomarkers in pSS, as well as of the epidemiologic, diagnostic, therapeutic, and prognostic aspects of this condition.

## Epidemiology

Cardiovascular diseases followed by malignancies and infections (7) represent leading causes of mortality in patients with pSS. Hematologic malignancies, namely B-cell lymphomas, are one of the most frequent causes of death in pSS, with an eightfold risk of mortality when compared with the general population. Non-Hodgkin’s lymphomas (NHL) occur in approximately 2.7–9.8% of pSS patients and recent data reported that NHL risk increases 2.2% per year of age with a 4.3-fold increased risk in pSS compared with the general population. With regard to the histotypes, MALT lymphoma, which accounts for 60% of cases, nodal marginal zone lymphoma (NMZL), and diffuse large B-cell lymphoma (DLBCL) are the most prevalent (4, 7). When considering other hematological cancers and solid tumors, data from a large, well-characterized cohort of Spanish pSS patients indicated that a small proportion of subjects (1%), developed non-B-cell hematologic malignancies, while the incidence of solid neoplasms was around 5%. In particular, an increased risk for thyroid, lip, oral cavity, stomach, and ovarian cancer has been observed (8, 9).

## Etiopathogenesis

Mucosa-associated lymphoid tissue lymphomas are associated with various chromosomal translocations that predominantly lead to increased activation of nuclear factor kappaB (NF-kB). NF-kB is a transcription factor that regulates many physiological and pathological processes mediating cytoplasmic-nuclear signaling. NF-kB activation is followed by its binding to DNA and modulation of the transcriptional activity of a wide range of genes (10). Chromosomal translocations are well-characterized tumorigenic events which, together with the chronic antigenic stimulation of autoreactive B cells, may explain the development of B-cell malignancies in pSS (11). Protein A20, a negative feedback regulator for NF-kB encoded by the gene TNFAIP3, is an ubiquitin-editing enzyme expressed in most cell types at low-basal levels. It is rapidly induced by tumor necrosis factor (TNF) or toll-like receptor-mediated NF-kB activation (12). Over 70% of pSS-related MALT lymphomas display mutations potentially leading to functional abnormalities in A20. To note, absent or weak protein A20 staining of lymphocytes was observed in in pSS minor salivary gland (MSG) biopsies and in the majority of lymphoma tissues carrying the mutations (13). Nocturne et al. reported an association between the rs2230926 exonic variant and an increased risk of lymphoma development. In particular, A20 functional abnormalities were observed in over 70% of pSS patients with MALT lymphoma. Half of these abnormalities displayed a germinal origin and half a somatic (tumor) origin. These results were confirmed in an independent cohort of European patients with pSS from the United Kingdom. These observations support a scenario in which the presence of germinal and/or somatic abnormalities of genes, leading to impaired control of NF-kB activation and consequent continuous stimulation of B cells, enhance the risk of lymphoma. Furthermore, they fit with the hypothesis of a “two-hit” process, in which a combination of germline and somatic abnormalities of TNFAIP3 promote the development of lymphoma (14–16).

B-cell activating factor (BAFF, BLyS) is a key molecule involved in the activation and stimulation of B lymphocytes and is a leading actor in pSS pathogenesis (17, 18). A mutation of the BAFF receptor, His159Tyr, is seen to confer an increased risk of lymphoproliferation in patients with NHL through activation of the NF-kB pathway (19–21).

This mutation was found in more than two-thirds of patients with pSS complicated by MALT and these patients displayed higher numbers of lymphocytes and a shorter interval to lymphoma development. Of interest, the presence of the mutation in pSS-non lymphoma patients was associated with hypergammaglobulinemia and rheumatoid factor (RF) positivity (22). Furthermore, the analysis of five single nucleotide polymorphisms (SNPs) of the BAFF gene (rs1224141, rs12583006, rs9514828, rs1041569, and rs9514827) in pSS patients with higher or lower risk of lymphoma according to clinical and serological features, revealed distinct genetic patterns. Recently, the haplotypes GTTC, TATT, TTCT, were identified and associated to disease susceptibility in pSS. In fact, these three reported haplotypes contain the risk alleles TC, TT, and CT of the rs9514828 and the rs9514827 SNPs, both located in the promoter of the BAFF gene. On this basis, the interaction of pSS-related BAFF gene haplotypes appears to contribute to the lymphomagenesis process (22).

**Figure 1:**
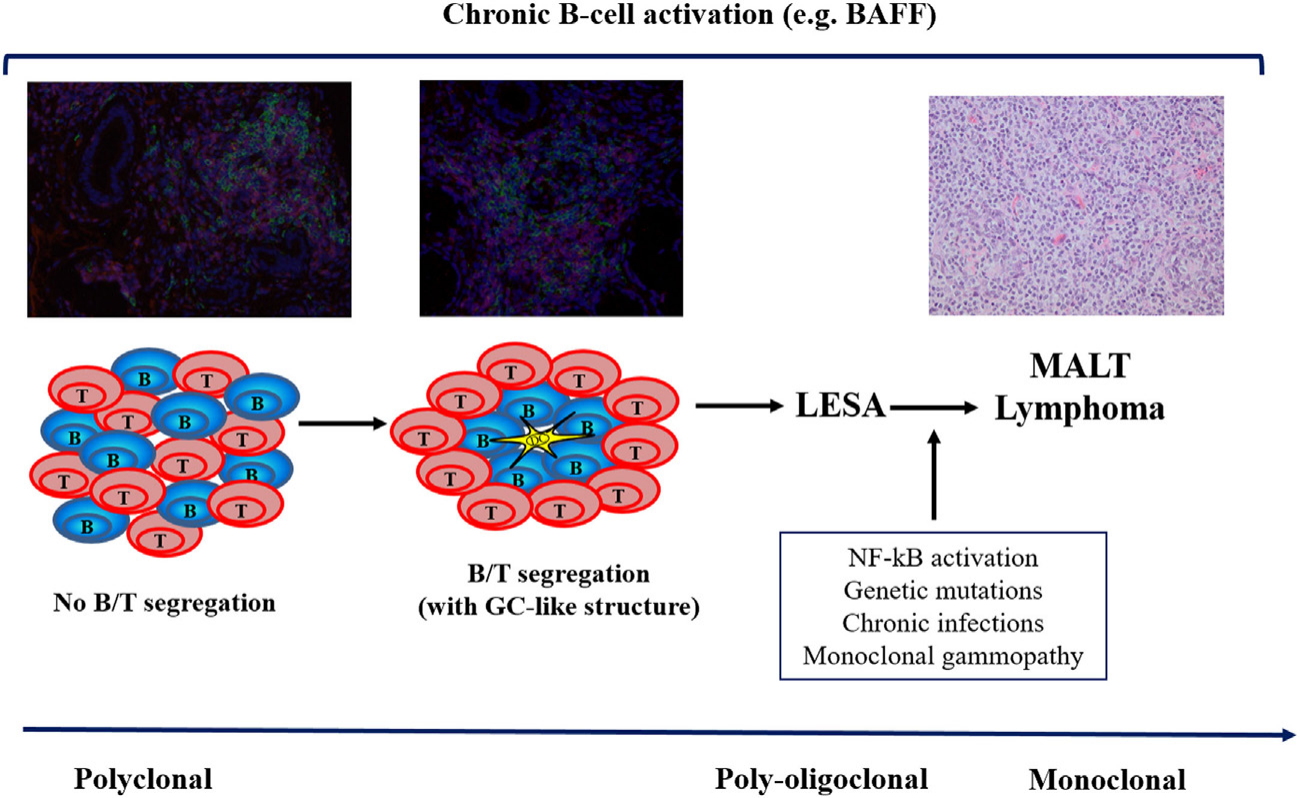
The multistep process of lymphomagenesis in primary Sjögren’s syndrome. BAFF, B-cell activating factor; GC, germinal center; LESA, lymphoepithelial sialadenitis; NF, nuclear factor; MALT, mucosa-associated lymphoid tissue.

Viral and bacterial infections inducing chronic site-specific inflammation have been significantly associated with MALT lymphomas arising in extranodal sites, including hepatitis C virus (HCV), Helicobacter pylori, Campylobacter jejuni, and Borrelia burgdoferi (23). Recently, the association between *Chlamydophila psittaci* (Cp) subclinical infection and marginal zone B-cell lymphoma of the ocular adnexa has been described. An Italian study demonstrated that Cp infection may also be involved in the development of salivary gland lymphoma during pSS, at least in a subgroup of patients. In fact, Cp DNA was detected more frequently in blood and tissue of pSS patients with MALT lymphoma compared with pSS patients with MESA or with no lymphoproliverative disease (24). Brito-Zeron et al. observed a prevalence of 13% of HCV in a large Spanish cohort of pSS patients. The lymphotropism of HCV suggests that the virus could act not only as a stimulus to produce and release cryoglobulins, but also to drive the overall lymphomagenesis process (25). The serological features of patients with pSS-HCV include the lower prevalence of anti-Ro and anti-La antibodies and higher frequency of cryoglobulins and related markers (25, 26) (Figure 1).

## Predictors of Lymphoma

### Histological

Besides the well-established diagnostic role of salivary gland histopathology in pSS, a growing number of studies put forward the hypothesis that specific histological features observed at the time of pSS diagnosis may predict the evolution of the disease. In pSS classification, the serological and histological criteria display the highest weight, but both need not necessarily be present in the same subject. This raised the discussion on whether the biopsy is really needed in patients fulfilling the serological criteria and having at least one objective evidence of sicca symptoms (27). The histological diagnosis of pSS relies on the observation of at least one focus, namely an aggregate of 50 or more mononuclear cells surrounding a duct or a vessel, within 4 mm^2^ of glandular tissue [focus score (FS) ≥ 1] (27). The FS can widely vary from 1, defined as above, to a situation where foci are confluent and spread throughout the glandular tissue, not allowing the observer to count them. In this case, the arbitrary score of 12 is given. Studies aiming at comparing the diagnostic performance of SG ultrasonography (US) and magnetic resonance imaging (MRI) revealed that both represent useful tools to identify structural abnormalities and display a good correlation with histopathology features (28).

However, the glandular inflammatory infiltrate is a dynamic environment; a more severe inflammation is associated with a different composition of the glandular infiltrate with T lymphocytes predominant in milder lesions and B cells dominating late stage lesions (29). Furthermore, the so-called ectopic lymphoid neogenesis, a process leading to the formation of germinal center (GC)-like structures, resembling those of secondary lymphoid organs, occurs in about 30% of patients (30). This large variability in the extent of glandular inflammation is not limited to the glandular side as it has been associated to a different clinical and serological picture (31, 32). In particular, patients with higher FS values display more extraglandular manifestations, including lymphoma (32–34).

Although different studies pointed out a major prevalence of GCs in MSGs or parotid glands of pSS patients that subsequently developed NHL (31, 35, 36), Haacke et al. recently ruled out an association between GCs in parotid glands and the development of lymphoma (37–39). At present, the relationship between GC-like structures and lymphoma is a matter of intense debate (37–43).

A set of recommendations has been developed with regard to salivary gland histopathology, however, there is still no consensus for the assessment of GCs, as several procedures are available but none has been proven to be superior to the others (44). In this regard, the comparison of the studies conducted so far, employing different methods to assess GC and using different tissues, namely minor or major salivary glands, is difficult and does not allow us to draw definitive conclusions (37–39, 42, 43). On this basis, imaging procedures are useful, however, they are not enough for a comprehensive evaluation of SGs and it is advisable to perform MSG biopsy in all pSS patients. This would allow for a clear picture of the status of the inflammatory infiltrate at the time of diagnosis and for the establishment of large cohorts to reach a consensus regarding the method to assess GCs and explore its prognostic value in more detail.

Type I and II interferons (IFN) have been associated with pSS pathogenesis and different patterns of type I and II IFN signatures have been associated with different clinical pictures (45). With regard to lymphoproliferation, the glandular IFNγ/IFNα mRNA ratio has been suggested as a potential biomarker of pSS-associated lymphoma (46).

A specific subset of B lymphocytes expressing the Fc receptor-like protein 4 (FcRL4) has been found to be expanded in parotid glands and associated with lymphoepithelial lesions (37–39). FcRL4 is normally expressed by lymphomatous B lymphocytes and the association of this protein with prelymphomatous conditions may support its pathogenic role in the process of lymphomagenesis.

A possible role of the glandular P2X7R-inflammasome axis has been postulated by Baldini et al. following the observation that such an increase is associated with the presence of GCs and is more pronounced in pSS patients that develop NHL (47). Although a number of other proteins, including cofilin-1, alpha-enolase, and Rho GDP-dissociation inhibitor 2 were found to be over-expressed in the salivary glands of patients with pSS or pSS/MALT lymphoma, further studies are needed to clarify their actual role in this scenario (48).

### Clinical and Serological

The first observation that specific pSS clinical features, such as parotid gland enlargement and lymphadenopathy, increase the risk of developing lymphoma dates back to the late 1970s (49). Several studies confirmed this finding and highlighted associations with other clinical and serological features (19–21). With regard to overall disease activity, it has been recently demonstrated that a stable moderate/high-disease activity, calculated either with the EULAR Sjögren’s syndrome disease activity index (ESSDAI) or with the ClinESSDAI, an ESSDAI variant excluding the biological domain, was independently associated with subsequent lymphoma occurrence (15, 16). In particular, while the activity in the lymphadenopathy domain at the time of pSS diagnosis was associated with any subtype of hematological cancers, activity in the glandular domain was associated to MALT lymphoma, and activity in the biological domain was associated with non-MALT lymphomas. Activity in the constitutional, pulmonary, and hematological domains was associated with non-B-cell cancers and poor survival (1, 2, 8).

Symptomatic cryoglobulinemic vasculitis (CV) is observed in about 3–4% of pSS patients and has been closely linked to the development of lymphoma (3, 50). Moreover, Retamozo et al. demonstrated that pSS patients positive for cryoglobulins and fulfilling CV classification criteria, had a higher frequency of type II cryoglobulinaemia, higher mean cryocrit levels and a higher cumulated mean ESSDAI score. After a mean follow-up of 110 months, 9% of patients developed B-cell lymphoma and 6% died. Therefore, the presence of CV at the time of diagnosis of pSS is independently associated with mortality and is strongly associated with higher baseline systemic activity (51). Quartuccio et al. confirmed this association with the ClinESSDAI (52). Interestingly, Risselada et al. observed that while peripheral neuropathy (PNP) and glomerulonephritis concurred with lymphoma in over 70% of cases, PNP was not associated with subsequent NHL development.

A recent Italian multicenter study aimed to identify biomarkers associated with lymphoproliferation in pSS patients with either NHL, CV without lymphoma, salivary gland enlargement without lymphoma or no CV, no salivary gland enlargement, and no lymphoma (controls). Cryoglobulinemia, low C4, anti-SSB/La antibodies, and leukopenia were significantly associated to lymphoma development. In patients with salivary gland swelling without NHL, negativity or positivity of only one of these biomarkers had a negative predictive value for lymphoma of around 90%. Conversely, positivity of at least 2 biomarkers identified a ninefold risk of lymphoma. No differences were observed with regard to positivity of the above-mentioned biomarkers in pSS with lymphoma versus pSS with CV but not lymphoma. This observation further supports the strong association between CV and lymphoma in pSS (50). As far as the association between leukopenia and lymphoma is concerned, it is still not clear whether leukopenia is an epiphenomenon of lymphoproliferation or a true predisposing factor for it.

The observation of high-BLyS levels in every pSS patient subgroup independently of the presence of a prelymphomatous condition or a fully blown lymphoma further supports the role of this molecule in every phase of pSS pathogenesis, including lymphomagenesis to induce and maintain B-cell survival.

Fms-like tyrosine kinase 3 ligand (Flt-3L) is a type I transmembrane protein also detectable as a soluble homodimeric protein, and able to activate Flt-3 to stimulate progenitor cells in the bone marrow and blood. Tobón et al. measuring serum levels of Flt-3L in 369 pSS patients, demonstrated that higher levels of Flt-3L were significantly associated with a history of lymphoma. In particular, an Flt-3L level of 175 pg/ml was strongly associated with lymphoma (specificity 97.5%, sensitivity 44%, negative predictive value 97%). Higher expression of Flt-3L was also associated with other clinical markers of lymphoma such as palpable purpura, low levels of C4, lymphocytopenia, low levels of IgM, high levels of β2-microglobulin, and a higher ESSDAI score. Since serum levels of Flt-3L were already increased in pSS patients at the time of lymphoma diagnosis, it can be speculated that high levels of Flt-3L may also be detectable in association with non-malignant lymphoproliferative disorder many years before the diagnosis of NHL. Among all of the previously described biologic markers, the Flt-3L concentration seems to be the most reliable as it is stable and not influenced by immunosuppressive drugs or biological therapy (53).

Tomi observed that approximately 10% of pSS patients with monoclonal gammopathy (MG) display a hematologic malignancy and that MG increases the risk of developing either MM or, to a lesser extent, lymphoma (54). Risselada observed that the presence of IgM-kappa clonal components was associated with lymphoma in 64% of cases (40, 41). In this setting, MG can be detected in about 7% of pSS patients and it has been associated with a higher ESSDAI and low-C4 level.

Lymphoid chemokines, including BAFF, and their receptors are crucial mediators for the activation of B lymphocytes and for the organization of the inflammatory infiltrate. Higher glandular levels of these molecules in pSS MSGs at the time of diagnosis compared with normal MSGs, or to MSGs with chronic non-specific sialadenitis have been already reported (31). In addition, pSS patients with lymphoma display higher serum levels of CCL11 and CXCL13 compared with patients without lymphoma (55, 56).

Fragkioudaki et al. developed a predictive model for the development of NHL including clinical, serological, and histopathological findings at the time of pSS diagnosis. Only clinical (salivary gland enlargement, lymphadenopathy, and Raynaud’s phenomenon) and serological (anti-Ro and anti-La antibodies, RF positivity, MG, and low C4) features resulted as independent adverse predictors for NHL development. Therefore, patients with two risk factors had a 3.8% probability of NHL development, while the probability of patients with three to six risk factors increased to 39.9%. If all seven risk factors were present, the probability reached 100% (57). Another study reported that ESSDAI and the international prognostic index (IPI) could represent valuable prognostic parameters of SS-associated NHL outcome (19–21).

Table 1 summarizes all of the above-mentioned predictors and corresponding references.

**Table 1:** Classical clinical, serological, and histological predictors of lymphoma development in primary Sjögren’s syndrome.

Predictive factors	References cited in the manuscript
**Clinical**	
Persistent enlargement of salivary glands	(19–21, 49, 57)
Lymphadenopathy	(19–21, 49, 57)
Symptomatic cryoglobulinemic vasculitis	(3, 50–52)
Peripheral neuropathy	(40, 41)
Glomerulonephritis	(40, 41)
Raynaud’s phenomenon	(57)
Stable moderate/high-disease activity, calculated with ESSDAI or clinESSDAI	(15, 16)
Concurrent chronic infections (Hepatitis C virus, Helicobacter pylori, Campylobacter jejuni, Borrelia burgdoferi, *Chlamydophila psittaci*)	(23–26)
**Serological**	
Leukopenia	(50)
Low C4	(50, 57)
Monoclonal gammopathy	(40, 41, 54, 57)
Cryoglobulinemia	(50, 51)
Autoantibody positivity (anti-SSA/Ro, anti-SSB/La, rheumatoid factor)	(50, 57)
**Histological**	
High focus score values	(32–34)
Presence of germinal centers	(31, 35, 36)

## Diagnosis, Therapeutic Approach, and Prognosis

As mentioned above, salivary glands are the most common site of MALT which, in the majority of cases, develops as parotid gland enlargement. Usually, MALT-related parotid gland enlargement is monolateral, persistent, fixed, and hard. Although the gold standard non-invasive procedure to differentiate between lymphoma and benign hyperplasia is MRI, US can be performed in the clinic in order to guide the biopsy procedure. A recent study demonstrated that dynamic contrast-enhanced-MRI could add important information for the differential diagnosis between MALT and benign hyperplasia (58). The histological features of MALT lymphomas include diffuse lymphoid infiltrates with small lymphocytes that may resemble centrocytes or plasma cells, which invade the tissue deranging glandular architecture. The demonstration of monoclonality using molecular biology testing allows for the differentiation of lymphoma from MESA/LESA, which are still oligo/polyclonal (55, 56). Usually, pSS-associated NHLs have an indolent course without B symptoms (fever, weight loss, and night sweats) and the treatment is related to stage at diagnosis, site(s), and histotype. Lymph nodes may or may not be involved. Localized low-grade lymphomas of the parotid gland may not require specific treatment but only active monitoring (59). Although no definite consensus exists for the treatment of parotid gland MALT lymphoma, immunochemotherapy is the most used approach for disseminated MALT, or localized MALT lymphomas of higher grade. A combination therapy with rituximab and either cyclophosphamide, chlorambucil, fludarabine, or bendamustine (60–62) has shown satisfactory outcomes. Patients with DLBCL should be treated with rituximab and the cyclophosphamide, doxorubicin, vincristine, and prednisone regimen (63). The addition of rituximab to conventional chemotherapy has shown major efficacy compared with chemotherapy alone (e.g., chlorambucil). Furthermore, chemotherapy-free regimens using only rituximab have shown excellent results. Therefore, it is advisable to tailor the therapeutic strategy according to disease and patient characteristics, whilst keeping in mind the pivotal role of rituximab alone or in combination with chemotherapy (64).

Overall NHL survival is currently estimated at 92% at 5 years, however, taking into account the ESSDAI and the IPI, a slight reduction (90.91%) was observed. In fact, patients with higher disease activity displayed a higher risk of relapse and death. When focusing on lymphoma subtype, MALT lymphoma displayed the best survival (94.12%) followed by NMZL (87.5%) and DLBCL (75%) (19–21, 65).

## Conclusion

Non-Hodgkin lymphoma is the most severe complication of pSS and occurs in around 5–10% of patients. MALT lymphoma is the most common subtype and is associated with a better survival rate. Several clinical, serological, and histopathological features have been proposed as predictive for lymphoma in pSS patients, allowing early diagnosis and consequently, better management and prognosis. In daily practice, clinicians should be alert to patients displaying clinical and serological features such as CV, low C4, and persistent parotid gland enlargement. On the other hand, the possible association between histological evidence of GCs in salivary gland tissue and lymphoma development remains to be defined and further studies are needed in order to draw definitive conclusions about the value of this histological assessment as an outcome predictor in pSS within routine clinical practice.

## Author Contributions

AA and FC conceived the idea of this review article. AA, ML, and FC prepared a first draft of the manuscript that was critically revised by RGe and RGi. All authors approved the final version of the manuscript.

## Conflict of Interest Statement

Authors do not have any personal, professional, or financial relationship that could potentially be construed as a conflict of interest.

## Abbreviations

BAFF, BlyS, B-cell activating factor; BAFF-R, BAFF receptor; CHOP, cyclophosphamide, doxorubicin, vincristine, and prednisone; Cp, *Chlamydophila psittaci*; CV, cryoglobulinemic vasculitis; DLBCL, diffuse large B-cell lymphoma; ESSDAI, EULAR Sjögren’s syndrome disease activity index; Flt-3L, Fms-like tyrosine kinase 3 ligand; FS, focus score; GC, germinal center; GN, glomerulonephritis; HCV, hepatitis C virus; IFN, interferons; IPI, international prognostic index; LESA, lymphoepithelial sialadenitis; MALT, mucosa-associated lymphoid tissue; MESA, myoepithelial sialadenitis; MG, monoclonal gammopathy; MRI, magnetic resonance imaging; MSG, minor salivary gland; MZL, marginal zone B-cell lymphoma; NF-kB, nuclear factor kappaB; NHL, Non-Hodgkin’s lymphomas; NMZL, nodal marginal zone lymphoma; OAML, marginal zone B-cell lymphoma of the ocular adnexa; PNP, peripheral neuropathy; pSS, primary Sjögren’s syndrome; RF, rheumaroid factor; SNPs, single nucleotide polymorphisms; TLR, toll-like receptor; TNF, tumor necrosis factor; US, ultrasonography.
